# Using CRISPR to understand how cancer mutations happen

**DOI:** 10.1038/s42003-021-02313-9

**Published:** 2021-06-23

**Authors:** Eve H. Rogers

**Affiliations:** Communications Biology, https://www.nature.com/commsbio/

## Abstract

Somatic mutations in cancer genomes can be caused by many different mutational processes, each of which produce distinctive patterns termed “mutational signatures”. Although cancer researchers can now recognize a large number of mutational signatures, exactly how these patterns arise remains unknown. Nik-Zainal and colleagues tackled this problem using a CRISPR-Cas9 genome editing screen to knock out components of the DNA mismatch repair machinery and learn their unique mutational patterns. Based on their data, the authors developed MMRDetect, a computational algorithm to classify the different DNA repair deficiencies and predict tumour responsiveness to immunotherapy.

**Figure:**
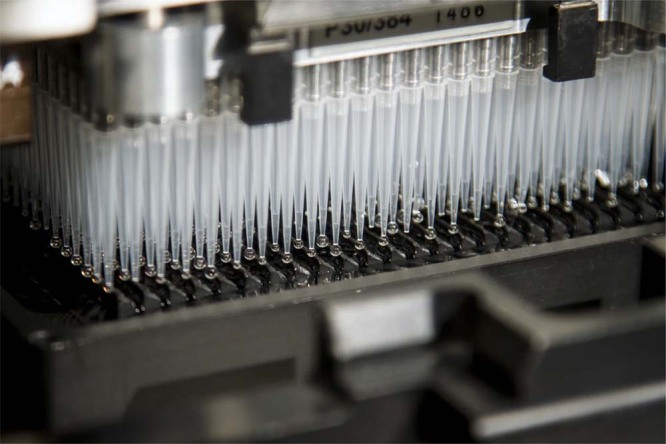
https://unsplash.com/photos/to8o0bqOA6Q

Somatic mutations in cancer often occur in recognizable patterns known as mutational signatures, which can be caused by external or internal processes. The challenge has been in teasing apart which mutations are a direct result of exogenous processes, like UV damage, and which are caused by a failure in the cell’s ability to repair endogenous damage, like that caused by oxidation or errors in replication. Understanding the consequences of specific repair pathway deficiencies could lead to improvements in personalised cancer treatment in the future.

Traditionally, mutational signature analysis has used cancer exome sequences, but there is an increasing desire to move towards analysis of whole genomes to identify greater numbers of somatic mutations and allow for signature decomposition. Zou *et al*.^1^ investigated the genome-wide mutagenesis consequences following DNA damage using whole-genome sequencing (WGS) data of colorectal cancers from patients recruited via the NHS-based UK 100,000 Genomes Project.

In their recent study published in *Nature Cancer*, Zou et al.^1^ combined CRISPR–Cas9 biallelic knockouts of several DNA replicative/repair genes in human induced pluripotent stem cells (hiPSCs) cultured in the absence of external DNA damage agents, to avoid confounding endogenous and exogenous sources of mutation. They generated 42 gene knockouts and conducted WGS of 173 subclones. The data were then compared to reported cancer-derived signatures, allowing the authors to identify nine DNA repair genes that produce mutational signatures implicated in DNA modification. Mechanistically, 8-oxo-2′-deoxyguanosine elimination was shown to be sequence-specific, whereas uracil clearance was found to be sequence independent. Mismatch repair (MMR) deficiency signatures were associated with oxidative damage and misincorporation. A reverse template slippage model was also suggested as a cause of DNA replication error and interestingly, ∆*MLH1*, ∆*MSH6* and ∆*MSH2* signatures were found to be similar to each other, but distinct from ∆*PMS2*. By directly mapping whole-genome mutations to DNA repair defects, these findings represent a step forward in understanding the replicative and repair genes key to genome maintenance against endogenous DNA damage.

Using their experimental data, the authors developed an R package called MMRDetect that can be used as a classifier to enhance the detection of MMR-deficient tumours in whole-genome sequenced cancers. Notably, potential applications include identifying tumours that could be sensitive to particular immunotherapies, as the identified mutational signatures could serve as biomarkers in precision medicine and identify targetable pathway defects. Current mutational assays can have reduced sensitivities in tumours with low proliferation rates, along with limited specificity. MMRDetect overcomes this challenge as it is based on experimental data and is shown by the authors to have enhanced sensitivity, particularly when detecting MMR-deficient samples with lower mutational burdens. MMRDetect could improve the detection and characterisation of tumour mutational patterns, but requires further testing to improve its sensitivity in other tumour types, beyond the current version trained on highly proliferative colorectal cancers.

Furthermore, another important consideration that must be noted is that the absence of a mutational signature does not necessarily mean that those genes do not transcribe important DNA repair genes, due to the effects of redundancy or compensation, missed detection of low rates of mutagenesis from background mutagenesis or the difference in responses of genes in vivo versus in vitro. These caveats must be considered before conclusions may be preemptively drawn. Therefore, MMRDetect allows us to gain an initial insight into the replication and repair genes that are key to DNA repair defects and paves the way for further investigation into this exciting area of research.
